# Living during COVID-19: qualitative analysis of Experts by Experience UK University Associates' socioeconomic inequalities, including readiness recommendations

**DOI:** 10.3389/fpubh.2025.1645667

**Published:** 2025-10-01

**Authors:** Joy M. Rooney

**Affiliations:** Independent Researcher, Birmingham, United Kingdom

**Keywords:** critical public health, equity in health, socioeconomical inequalities in health, disability, experts by experience, COVID-19, hidden disabilities, vulnerable group

## Abstract

A vulnerable group of people with disabilities and carers (Experts by Experience) wished for research during the COVID-19 pandemic to assist other people with disabilities in the future. Consideration of this vulnerable group seems to be limited in future policy pandemic readiness despite huge potential mortality. Literature reviewed critical public health theory through assessing health inequalities and health equity in relation to disability/chronic ill health. A standard qualitative thematic method distinguished socioeconomic health inequalities for people with earlier mental/psychosocial distress and other Experts by Experience-including people with physical, visual, self-identified hidden disabilities, and their carers. Environmental, community, and individual challenge were part of the overarching theme. Up to five sub-themes were: shielding, diminished quality of life, use of social media vs. the digital divide, people with hidden disabilities, activism, celebrating lockdowns' ends, loss in stability of outlook, loneliness/isolation, coping strategies, developing new skills, re-evaluating lives because the world will change. Discussion of findings relied on four critical public health pillars; included were future recommendations for pandemic/emergency readiness.

## 1 Introduction

Globally, including the UK, the viral COVID-19 pandemic or syndemic affected vulnerable or bioprecarious groups of people through hugely disproportionate mortality, and potential microbial resistance to antibiotics continues (1–6). Such vulnerable groups contain individuals or communities experiencing for example, homelessness, poverty, disability, mental illness, substance use/addictions, physical illness, young or old age and/or ethnicity, race or Indigenous (3, 7–9). The World Health Organization defined disability as individuals with impairment in body structure or function, or mental function (for example, loss of a limb, loss of vision or memory loss), activity limitations [such as working, engaging in social and recreational activities, and obtaining healthcare and preventative services, World Health Organization (10)]. In the UK, people Clinically Extremely Vulnerable (CEV) with chronic ill health were increasingly identified four times as communication improved. They were requested to shield through Government letters asking them to stay at home. People with disabilities, including mental health, and CEV had the highest mortality numbers (11). Yet in the UK the Office for National Statistics (ONS) was only able to report on these vulnerable individuals following death (12). The most recent World Health Organization Disability Summit resolutions included disaggregating surveillance methods to identify susceptible people with disabilities within 5 years.

While there is much critical public health theory, more recently Schrecker (13) relied on five intersecting and interacting pillars. Rearranged these are:

- History and legacy.
- Health inequalities result from underlying social arrangements or institutions.
- Over-medicalization and the dominance of medical frames of reference.
- Commitment to health equity.
- Socially driven increased scientific knowledge governed by materials and institutes.

The first four pillars will be considered in relation to people with disabilities.

Chen and Wang (14) and Hanson et al. (15) reported people with disabilities held least assets/social economic status, and so lost wellbeing due to ongoing austerity of Governments through freezing/reducing benefits, health and social care. These authors recommended that nations should understand people with disabilities to support their self-perpetuating recovery. In the UK the practiced (Exercise Cygnus), influenza preparedness strategy was initially implemented by government, however, was not designed for a more virulent virus (16–18). Rietveld et al. (19) concentrated on reporting UK preparedness and early response January 2020-March 2020 finding it woefully lacking. While the British Medical Association (BMA) Report Four, the Public Health response of Government, was intensely critical (5). BMA Report Five concentrated on reporting poor population health due to austerity prior to the pandemic and inequitable groups during the pandemic, including those people with disabilities (6). Effects of COVID-19 on people with different impairments (learning disabilities, hearing and mental health) were distinguished (6). Within the 1st year of the pandemic it was debatable whether authoritarian and neoliberal principles or the public health of social democracy were employed by the UK government (20). In the UK, decision-making conflicted between central government interference, and earlier local authority emergency civil planning (21). Was there censorship of information, or was accessible information not aligned (22, 23)?

Underlying social or institutional arrangements facilitated health inequalities for those with disabilities (24). The disability/CEV/chronic illnesses UK mortality was 58% (11). Included in this figure was the 2.5 times increase in mortality of people CEV compared with a matched general population during shielding (25). Yet globally, this vulnerable group is 16% of the worldwide population (26). Beresford et al. (27) and Duffy et al. (28) recommended that any research should include people CEV. Analyses of NATO nations' responses to protect this differentiated population suggested they were inadequate, not reducing reported mortalities (29). In Wales, UK the shielding populations' mortality was high (30). Governments did not consider people with hidden disabilities as any priority during the COVID-19 pandemic, including people with earlier mental distress, and visual challenges (not using any aids (31, 32)). Although much later, it was suggested that people with disabilities, although challenged, showed great resilience to the COVID-19 pandemic (33).

Initially, people with disabilities/CEV/chronic illnesses/shielding may have never known understood/implemented any, or all safe practices to keep living, for example, hand washing, isolating themselves from others, and the right to be tested for COVID-19 because no targeted communication/provision of information, based on special need resulting from different disabilities, was provided by, for example, the devolved UK governments. Later, such people again had no differentially targeted communication/provision of information and guidance regarding face mask wearing/PPE for themselves/carers, social distancing when leaving home, and receiving vaccinations as priorities (34–37).

People with disabilities were disregarded and ignored due to an inhuman belief in “normalcy” (38, 39). Nations favored citizens without disabilities, who formed an ableist majority for survival (40, 41). To date literature reviews concentrated on people with disability or types of impairment, those with CEV/chronic illnesses, occasionally distinguishing conditions (33, 42–44). Barriers were cited as changes to care and rehabilitation, mental health impact, access to information, financial impacts, ease of communication, access to essential services, educational challenges, and physical safety (42) While facilitators were new innovations, changes to care and rehabilitation, familiar and social support and inclusive policy changes (42). Lombe (44) highlighted violence experienced by individuals with disabilities, unequitable healthcare for those with pre-existing mental distress and those people disabled through visual challenges, and non-inclusivity of those who were disabled. Beevi et al. (33) took a completely different stance suggesting disabled people were supported by governments, authorities and were resilient to their challenges. Holm et al. (45) statistically significantly linked psychological distress of people with mobility, vision, hearing, cognitive, and any others to increased sleeping problems/nightmares, and reduced daily exercise for people with mobility challenges. The COVID-19 pandemic caused concern for guide dog owners' health, reducing their mobility by 83% (46). In addition, COVID-19 reduced work, study, exercise, volunteering, hobbies, other activities, and staying connected with others (46). People with earlier mental distress undertook less exercise during the first lockdown (47–50).

Although it may be thought in critical public health theory over-medicalizing occurs, people with disabilities may require increased medical interventions. Across the world people with disabilities had poorer access to healthcare services (51, 52). The first wave of COVID-19 pandemic affected people with chronic conditions psychologically, and therefore they required rapid support (53, 54). Most people who shielded with earlier mental distress, were more likely to have poor mental health, and life satisfaction during the pandemic, so demonstrating perpetuation of existing mental health inequalities, and social injustice (55–58).

Equity in health is the absence of unfair, avoidable, or remediable differences among groups of people, whether those groups are defined socially, economically, demographically, or geographically, or by other dimensions of inequality such as sex, gender, ethnicity, disability, or sexual orientation, or race, ethnicity or Indigenous (59) Providing equity may result in equality under the UK Equality Act (2010). Clearly, as an ideal, equity might ensure fair distribution and implementation of public health interventions in preparedness driven by the public health needs of differing population groups (59). However, from recognition of a COVID-19 global pandemic, world governments concentrated on protecting themselves, their countries' economies, health and social caring professionals, and supporting the majority. *Prolonged duration of this pandemic enabled a limited number of governments to enact* the Rights of Persons with Disabilities (UNCRPD) (60). In fact, most nations, including the UK, did not successfully implement human rights to life, health, education, work, standard of living, and community for people living with disabilities. This was due to unpreparedness, through holding insufficient knowledge of complex socioeconomics, despite organizations such as the international disability movement creating “invited and invented space” (61). The disabled rights movement of people with disabilities-led third sector organizations, supporters, allies, and academics, maintained campaigning activities including social justice, rights-to-life/equity, and co-produced research of this marginalized group during the COVID-19 pandemic. Most of these people used new, remote, online, social media platforms, despite their challenges (27, 62). Such UK organizations included, for example, Disability Rights UK,^1^ Comensus,^2^ Shaping Our Lives,^3^ National Survivor Users Network,^4^ Centre for Mental Health (63), and Gilbert (64). These organizations, and individuals continued to campaign for appropriate policies, and procedures before, and during the UK Public Inquiry^5^ (28, 65). Cullingworth et al. (66) reported on much supplementary, complementary and adversarial involvement of multiple UK Disabled People's Organizations in stepping in immediately the pandemic was apparent. Interactions between Government agencies and the UK Third Sector were more complex than a demand supply model, rather it was as a reflection and enabler of civil society, building and mobilizing social capital (66). From March 2020, two struggling third sector organizations in Ontario, Canada demonstrated grass roots “essential” disability justice in contrast to discrimination of authorities against disabled people (67). In USA it was not until September 2021 that partnerships with disability-led organizations were formed for emergency response and vaccination planning (68).

Equity includes *s*ocioeconomic challenges which were through poverty (economics/access to government payments/finances/debt); exclusion from education and employment/ability to work; access to digital/IT technology, and barriers faced within the health system (69–72). Any workers with zero hour contracts who were unable to work during COVID-19 were not eligible for any UK Government grants, including those Experts by Experience in the current study, and overseas (41). In fact, in Germany during COVID-19, subjective wellbeing was most significantly demonstrated to be affected by those with disabilities and chronic illnesses rather than socioeconomic status (73).

Many belonged to the “digital divide”, and therefore upskilling in the use of the Internet, and social media was challenging due to poor accessibility within programs on their devices, including mobile phones, non-available training and inadequate personal funds (74, 75). Social media use through Internet connectivity during lockdown was associated with increased anxiety, or anxiety and depression in China (76). It also exacerbated sleep difficulties, paranoia about acquiring COVID-19 infection, distress, and panic in South Asian nations (77). However, there was also potential benefit to people with disabilities, and their carers, through increased contact with others during COVID-19 isolation, across the “digital divide” (78).

Socioeconomic determinants of public health including environmental, community, and individual challenges resulted in exclusion, loneliness, isolation, poor coping strategies, and suicide in some individuals (57, 79–89). Yet in others, overcoming such barriers were through developing coping strategies to stay-alive/well, continuing to work, modifying lifestyles, for example, ensuring communication/social bonding/relationships with others (family, friends, third sector organizations, professionals), keeping busy, exercising, undertaking hobbies/interests/activities, and study/education (46, 47, 50, 57, 72, 90–98). Kahlon et al. (99) identified lay-people as potential beneficial telephone advocates during the pandemic. Clearly, all individuals with disabilities/CEV/chronic illnesses/shielding had a wide spectrum of character strengths/positive psychology, irrespective of health challenges (9, 100). Indeed in the USA, subjective wellbeing in people with reduced activities of daily living was increased with social support (101). However, those most severely limited also might require increased instruments (physical and financial resources) during a future pandemic (101).

People with disabilities live within communities, and environments distinguished by their socioeconomical challenges. Community challenge for people with disabilities was reported to be inclusion to live day to day, with equity (69, 71, 72). In addition, people with disabilities often were abused, stigmatized and discriminated against by other citizens (92, 102–104). Many environmental challenges were reported to be through inaccessibility/emergency prohibition/lack of prioritization, for example, inaccessibility of shops to buy food, GP surgeries/mental health/other health services/medication, and COVID-19 vaccinations/social services/support/respite care, transportation/buses and public buildings for financial support/disability benefits, and employment support (45, 69, 92, 96).

To alleviate such enormous, future mortalities The UK COVID-19 Public Inquiry^5^ seeks to include all stakeholders. It seems those people with disabilities and CEV form a very small proportion in their deliberations compared to such huge mortalities (105). How will it ensure equity in accessing health for those vulnerable people with disabilities and CEV through stakeholder participation and social justice, employing public health interventions socio-economically? Certainly, the UK's most recent policy Green Paper “Health and Disability Benefit Reforms” predictions suggest reduced disability benefit payments for most adults, with 1,000 more work coaches employed to encourage those with disabilities to enter the workforce (106).

The World Health Organization stepped down COVID-19 pandemic status during the first week of May 2023 (107). Partners across the world continued to work together through the International Pandemic Preparedness Secretariat (IPPS) to promote a global availability of diagnostics, therapeutics, and vaccines within the first 100 days of a pandemic threat (108). A World Health Organization Pandemic Agreement was adopted by member states at the 78th World Health Organization Summit during 2025 for proposed global health governance during future pandemics to ensure strengthened pandemic prevention, preparedness and response (109). Although Lazarus et al. (110) suggested that the World Health Organization remains challenged because sufficiently clear compliance, accountability, and enforcement mechanisms are not present.

In the UK, recently, updated guidelines for emergency responders within Local Resilience Forums were published for identifying and supporting people who are vulnerable in an emergency (111). How to identify those people with hidden disabilities was not included. Case studies for data sharing arrangements, and Community Champions were mentioned, and exercise TOLLARD materials are available for practicing emergency responses for vulnerable people, where vulnerable was defined as “people who are less able to help themselves in the circumstances of an emergency, who must be given special consideration in plans.” [108, p. 7]. Accessible disabled persons advice was prepared through consultation with all stakeholders and is kept under review (112). However, not all people with disabilities will find this online communication method accessible. The UK Office of National Statistics risk vulnerability tool for people with disabilities will be employed during a national large scale pandemic response exercise during Autumn 2025 (113). It was reported to allow identification proportionately impacted disabled groups ahead of and during crises, and enable targeted local support where required (113). From the beginning of the UK COVID-19 pandemic, Experts by Experience, with lived experience of disabilities, many of whom were also CEV/chronic illnesses, and their carers, as associates of a UK university (named IMPACT), met using an online platform. They communicated experiences to each other and provided mutual support (Experts by Experience who were visually challenged opted out). A research idea to benefit other people using their insights to stay alive during the current COVID-19 pandemic formed.

The research objective included the socioeconomic effects of COVID-19 on a group of university Experts by Experience, a year apart, nearing the end of the first and third UK lockdowns.

The Experts by Experience in this study had such disabilities or were carers of those adults who did. Their ages were below those who might experience disabilities, for example, in the UK, adults, 14–25 years before death (114). Their insights were important when critiquing power imbalances in capital, medicine, availability of commodities, and equity. The wishes of these Experts by Experience to make a difference were conducted by an experienced academic, and survivor researcher who was also an Expert by Experience associate of this university. UK national, and local actions toward equity in health access of this vulnerable group were not thought sufficient. Although all were thankful to remain alive possibly due to devising their own coping strategies and mutual support provided. The outcomes of this study were used to generate ideas for recommendations to enable preparedness for another pandemic, especially during the first 100 days following declaration.

## 2 Methods

### 2.1 Study design

An academic researcher/survivor researcher/Expert by Experience associate, and IMPACT facilitator (a principal lecturer in social work) reached out to invite all Experts by Experience associates by e-mails (*n* = 23; where n was the total number of active Experts by Experience associates, minus the researcher) to join a mixed methods study. A survey monkey generated and collected results from anonymous participants; each allocated a different code number. A pocket voice recorder recorded telephone interviews, 30–60 min in length. Semi-structured interview questioning of Experts by Experience concerned:

1) Perceived changes in mental health; also determining whether an Expert by Experience specialized in mental health due to lived experience.
2) How time was spent at home during lockdown.
3) How connection with others was achieved, including use of social media.
4) Contributions made through undertaking new roles/developing new skills.
5) Perceived long-term effects because of COVID-19, even if Experts by Experience remain virus-free.
6) Lasting changes to be made for the future.
7) Celebrations planned at end of lockdowns.
8) Any other comments.

In addition, unused research outputs from a 2015 study regarding the voices of similar self-selecting Experts by Experience facilitated comparison of activism, pre- and during the COVID-19 pandemic.

### 2.2 Participants

Experts by Experience were people with an expectation of equal relationships between health and care professionals because they used health and social care services due to their disabilities, the Clinically Extremely Vulnerable (CEV), or carers of such people; they had training and practice [adapted from McLaughlin (115)]. Involvement of Experts by Experience was mandated in English Universities by the regulatory bodies of professions taught, for example, Social Work (116), Nursing and Nursing Associates (117, 118), Occupational Therapy, Physiotherapy, and Paramedics (119). Recommended but not mandated was Experts by Experience involvement in the training for Physicians Associate whose profession body is the Royal College of Physicians (120).

Thus, for more than 10 years, this university had Expert by Experience associate staff members awarded 0 h contacts. Their paid tasks were student/staff selection, sharing personal stories/narratives of individual disabilities resulting in health interventions and social care support, preparing/delivering complete teaching/assessment sessions within university student teaching modules, sitting on university committees, original research, and validating course content.

Most Experts by Experience received Royal Mail letters from the UK government advising shielding for twelve weeks during the first UK lockdown because they appeared on medical databases classified as CEV. The researcher interviewed Experts by Experience toward the end of the first UK lockdown in May 2020, and third lockdown during May 2021; each sampling took place over a time interval of 1 week. No demographic information was collected because all Experts by Experience lived in one region of UK, and there was a risk of identification. Ten Experts by Experience self-identified their disability as earlier (pre-COVID-19) mental/psychosocial distress, for example, bipolar, post-trauma(s) (PTSD), obsessive compulsion(s) (OCD), anxiety, depression, psychosis, and anorexia. Six other Experts by Experience self-identified their disabilities to include people being visually challenged, a person who was a wheelchair user with cerebral palsy, and CEV/chronic ill health survivors, for example, a person living following stroke, with diabetes, and people caring for family members with mental/psychosocial distress or learning disabilities. During 2021, a single Expert by Experience, self-identified with earlier mental/psychosocial distress declined a second interview. Two other Experts by Experience also volunteered; another person was visually challenged, the other person had chronic obstructive pulmonary disease (COPD). Therefore, there were 16 anonymous respondents interviewed in 2020 (designated an anonymous number), and 17 in 2021 (designated an anonymous letter) resulting in a total of 33 telephone interviews including 18 Experts by Experience.

### 2.3 Procedures and data collection

The design ensured all participants were able to participate despite their disabilities. The researcher coded each interview differently to ensure anonymity between years, uploaded to the university's cloud system, for subsequent transfer, and transcription by an independent transcribing company via a secure drop-box. This company returned transcriptions, password protected, for analysis.

A total of 18 anonymous respondents completed an online survey, however, very few people participated in the 2021 survey, possibly because a single email invited participants to both telephone interview, and online survey simultaneously. This researcher discounted online surveys in both years because most Experts by Experience self-identified as experiencing earlier mental distress in the 1st year. Therefore, linking the two methods of sampling proved to be impossible, and more insights were gained from interviews.

### 2.4 Data analysis

The researcher electronically stored, printed and qualitatively thematically analyzed interview transcriptions through step-by-step guidelines (121–128). Interview transcriptions were analyzed through independently coding interesting features of the data systematically across the entire data set, collating data relevant to each code, and then counted (coding and electronic transcription “find navigation”) four times to ensure accuracy, with no bias. Emergent themes, and sub-themes, containing commentary and verbatim quotations, suggested that participants occurred as two sub-groups: Experts by Experience self-identifying with earlier mental/psychosocial distress, and others. Others included carers and those with impairments in sight, limbs, physical illnesses and CEV/chronic illnesses. Those with hidden disabilities were self-identifying and included those people with disabilities in mental/psychosocial health and those visually challenged.

### 2.5 Ethical considerations

A UK University Ethics Board (reference CHLES) granted ethical approval during 2020, including an extension during 2021. During 2015 this university also granted ethics approval for earlier, mostly published research by this author. Thus, all procedures contributing to this study complied with the ethical standards of the relevant national and institutional committees on human experimentation, and with the Helsinki Declaration of 2008.

## 3 Results

Insights of Experts by Experience produced the following key themes: health inequality/inequity in socioeconomics, with interconnecting environmental, community and individual challenges. There were up to five sub-themes emerging for each interconnected theme– shielding, diminished quality of life, use of social media vs. the digital divide; people with hidden disabilities, activism, celebrating lockdown's ends; loss in stability of outlook, loneliness/isolation, coping strategies, developing new skills, re-evaluation of life because the world will change.

### 3.1 Socioeconomics

All those interviewed had no income streams from their work as Experts by Experience during the first and third UK lockdowns of the COVID-19 pandemic, so relied on earlier UK Government benefits to continue living. They were not eligible for finance from UK Government grants or subsidies during this period. However, most were eligible for weekly Government food-boxes.

#### 3.1.1 Shielding

Of the 18 Experts by Experience who participated in this study, most were shielding toward the end of the first UK lockdown in May 2020, and many were continuing to shield a year later, toward the end of the third lockdown (May, 2021). This was despite advice letters for the Clinically Extremely Vulnerable (CEV) sent by UK Government during lockdown one, suggesting shielding for a 12-week duration. An example of a quotation from an other Expert by Experience who was CEV, shielding for longer that Autumn 2020 was:

“*Yes I did because I went for a long, long period without meeting anyone. I do recall, particularly the first lockdown March 2020, it was a full 12 months after that, I had no contact with anyone”* (other Expert by Experience 2021 E).

None of the participants contracted COVID-19 during this study.

#### 3.1.2 Diminished quality of life

During the first lockdown, all participants were dependent, in different ways, on health and social care, and they perceived that their quality of life had diminished through, for example, the majority had cancellation of all NHS appointments, the majority had difficulties obtaining food and prescriptions when shielding. One example, provided by an expert with experience with earlier mental distress who became unwell during the first UK lockdown, demonstrated how health professionals had to enforce social distancing, not seeing anyone in crisis:

“*I think the mental health team, although they were in lockdown as well, I think not having face to face contact in crisis is quite difficult. Because the guy who came round and dropped the diazepam, he just put it on the windowsill.”* (person with earlier mental distress 2020 9).

All Experts by Experience mentioned ongoing reduced quality of life toward the end of UK lockdown three, however, they accepted its “normalcy”.

#### 3.1.3 Use of social media vs. the digital divide

With isolation during all lockdowns, use of social media was hugely important to most Experts by Experience. Some spoke out regarding making new contacts, mutual support, and the advantages of online communication. Two examples of quotations from both groups of Experts by Experience demonstrated their positivity:

“*Working remotely has been a godsend for me because my mobility has got a lot worse.”* (person with earlier mental distress 2021 J).

“*I think social media has been a good thing. I have wondered if I'd have maybe felt more isolated and lonelier if I hadn't have been able to have that contact.”* (other Expert by Experience 2021 O).

However, an equal number of others chose to disengage into the digital divide. Their reasons included: destructive forces of misinformation, tiring sitting at a screen, and missing clues about people when not engaging in person/in venue. Examples of quotations of such negative beliefs from both groups suggested that:

“*It can be perhaps destructive, because you miss out on all sorts of other cues…But I find it very tiring, I much prefer that face-to-face contact”*. (other Expert by Experience 2021 L).

“*I don't use social media, I decided to disengage myself from it about six months ago, and I feel a whole lot better for having done so.”* (person with earlier mental distress 2021 M).

### 3.2 Environmental

#### 3.2.1 People with hidden disabilities

Several Experts by Experience spoke out about how the public were treating them toward the end of the UK 2021 lockdown while they were navigating their local environments. Their voices highlighted lack of sympathy, support, and verbal/potentially physical abuse. People with hidden disabilities fared the worst, for example, with transportation, a person with earlier mental distress traveling on a bus being verbally, and potentially physically abused:

“*I have a real worry about that, I've been sworn at, I've been threatened before because I've been sitting in a disabled seat when I happen not to have a walking stick with me. And I thought I was going to get beaten up, and the driver did absolutely nothing about it, and I just don't feel safe.”* (person with earlier mental distress 2021 J).

Secondly, with ever-changing signs regarding navigation within local food shops, a visually challenged person (not using any aids) believed they had no hope of success:

“*if you've got a visual impairment just because of the non-contact, the social distancing, any support you might need. If …you made a bit of a mistake, it's not that easy to stop someone,… can you just help me out a bit with navigation?* (other Expert by Experience 2021 E).

### 3.3 Community

#### 3.3.1 Activism

More grass roots activism within statutory and/or community third sector organizations occurred pre-COVID-19, with less activity by all Experts by Experience during lockdown one. Pre-COVID-19 paid work at a different university, or within professionals' organizations, was greater with people with earlier mental distress compared to other Experts by Experience. In addition, there was more face-to-face volunteering by the majority of other Experts by Experience compared to people with earlier mental distress. There was more telephone support of people with disabilities by the majority of other Experts by Experience compared to people with earlier mental distress at the end of both lockdowns. However, a few Experts by Experience with earlier mental distress led community organizations during lockdowns.

Pre-COVID-19, two examples of quotes by Experts by Experience who were either visually challenged, or had earlier mental distress, demonstrated both communities, and universities benefited from each other:

“*I'm a trustee of Sight Concern, which is the local sight loss charity, and I think some of my experience at the university spreads just by being there, walking in and out, with a dog…*” (participant 3 2015).

“*Well, my involvement with the police work would be as an independent advisory group member definitely benefited, because I think they cross over, because again the police is education or best practice sort of thing.”* (participant 19 2015).

#### 3.3.2 Celebrating lockdowns' ends

All participants viewed returning to universities' work in the community, following the third UK 2021 lockdown as celebratory. However, some Experts by Experience viewed the end of both COVID-19 lockdowns as not the time to celebrate in any other way, due to their financial circumstances. Indeed, celebrations by most involved friends and family, travel and holidays:

“*Well, I think very soon after that the family will be getting together as a big group.”* (other Expert by Experience 2021 K).

All other Experts by Experience were more likely to be meeting up for meals out, and at coffee shops only following the first lockdown. Other celebrations included being with real live people again, returning of carers' respite, completing a sports management course and applying for continued funding of a community-based mental health support group.

### 3.4 Individual challenges

#### 3.4.1 Loss of stability in outlook

It was noteworthy that only half the other Experts by Experience reported any issues in loss of stability in outlook during the first 2020 lockdown. They spoke of, for example, boredom, and increased stability/productivity following on-line meetings' interactions. Most people with earlier mental distress believed their mental health was in decline following 10 weeks of lockdown, especially during the 2 weeks prior to interview. Other Experts by Experience who were visually challenged had lost confidence. They were anxious and panicky about going into all shops due to the social distancing requirements and not being able to follow protocols. Voices of loss in stability by all Experts by Experience toward the end of lockdown one in 2020, in order of frequency included: worry, anxiety, awareness of COVID-19, frustration, concern, feeling locked up, strange, doomed, panic, anger-threatened by the virus, annoyance and solidarity/affinity with others.

These following three quotations provided examples of declining mental health and substance misuse by an Expert by Experience with earlier distress, or no effect on mental health of an other Expert by Experience, both provided during the first UK lockdown. The third quotation following a year of the COVID-19 pandemic from an earlier mentally distressed Expert by Experience demonstrated continued poor mental health:

“*Yes, I do consider it has changed. I consider I've changed for the worse, and I've become aware, certainly over the last two or three weeks that my mental health is going downhill somewhat… So I will drink over the weekend and then sometimes I'll have a bottle of rum as well.”* (person with earlier mental distress 2020 6).

“*I don't think that my mental health has changed…I don't think that any of us can be entirely free from the worries and concerns of what COVID-19 might bring and lockdown and the consequences of that..”* (other Expert by Experience 2020 11).

“*Yes, I would say that I have had experiences of being much more depressed and anxious than I would have been without it..”* (person with earlier mental distress 2021 J).

Near the end of the third UK lockdown COVID-19 in 2021 the mental health of several other people with earlier mental distress reported they were no longer affected. In contrast to the last quotation, the voice of one Expert by Experience with earlier mental distress, regarding their ability to withstand adversity through trauma and hardship was noteworthy:

“*I feel that some of the most, people who've been the most resilient to the challenges of lockdowns and the pandemic have been people with pre-existing mental health, because we've already experienced such trauma and hardship in certain ways that have made us mentally in the first place.”* (person with earlier mental distress 2021 I).

Most Experts by Experience considered loss of stability in outlook, was less nearing the end of 2021 UK lockdown, excepting the frequency that they mentioned frustration, and anger.

#### 3.4.2 Loneliness/isolation

For many participants living alone, all other Experts by Experience spoke of loneliness. However, many people with earlier mental distress did not believe they were lonely near the end of lockdown three in 2021. An example, provided by an Expert by Experience with earlier mental distress demonstrated his acceptance of being alone. This quotation demonstrated that such people were already socially isolated:

“*I think I am isolated. I'm certainly alone, and most of the time I don't mind my own company, in fact I need it.”* (person with earlier mental distress 2021 M).

Descriptions of isolation were indistinguishable between the two groups of Experts by Experience.

#### 3.4.3 Coping strategies

A hierarchy of coping strategies included activities to alleviate more home-spent-time, for example, regular physical exercise, using on-line media platforms, reading more, cooking and baking more, gardening, planning and being organized, staying connected with others, watching TV more, arts and crafts including repairing, spring cleaning, and sleeping/drinking more alcohol. For all other Experts by Experience there were more limited activities at the end of the first lockdown. However, such differences disappeared during the third UK lockdown in 2021. Although most other Experts by Experience undertook more exercise than a few people with earlier mental distress during the first lockdown. Between 2020 and 2021 all Experts by Experience with earlier mental distress increased their physical exercise. Therefore, any initial difference had disappeared by the end of the third lockdown.

Use of on-line media platforms was much higher for all people with earlier mental distress. Many from both groups of Experts by Experience read more during the first lockdown which diminished during the third lockdown. Cooking and baking was higher in other Experts by Experience at the end of the first lockdown. In contrast, gardening appeared more popular for people with earlier mental distress during the first lockdown. There were similar levels of both these activities across both groups during the third lockdown.

There was an indication that most Experts by Experience with earlier mental distress watched more TV and undertook more arts and crafts activities during the first lockdown. In addition, this group undertook more spring cleaning, sleep and alcohol consumption. Several other Experts by Experience mentioned household chores/tasks more often in both years. A wide range of other ideas for coping strategies with lower frequencies included: DIY and decorating, carrying on as normal, quizzing, eating, doing tax return, getting supplies/medications, song writing, playing golf, undertaking pet care, developing IT skills, developing indoor exercises, clapping for NHS and meeting neighbors, erecting garden fence, tutoring, undertaking word searches, making a band video, attending on-line church services, studying, and passing qualifications.

A minority of all Experts by Experience demonstrated their ability to procrastinate, through making unachieved to do lists during lockdown one, and feeling continuously bored through all lockdowns. Examples of quotations were:

“*I'm very good at making lists of things that I could do during lockdown, and I've not achieved many of them.”* (person with earlier mental distress 2020 2).

“*Well, I'm still very bored, because I was going to the gym before I think, but then they locked down obviously so I couldn't go.”* (other Expert by Experience 2021 C).

#### 3.4.4 Developing new skills

Understandably, all Experts by Experience developed more new skills between the first and the third lockdowns in 2021 compared with the first lockdown during 2020, because of differing time durations. These included by frequency: the majority of other Experts by experience social media platform learning, crafting, learning indoor exercises, making damson gin, editing photographs, starting a new job from home, increasing interview techniques, checking text for accuracy (paid word at home), learning how to negotiate online, undertaking a holiday role, learning pet care, planning adaptations for home, learning through webinar workshops, reading/scoping, learning physical skills to care more. A few other experts by experience obtained new qualifications, increased speaking and listening skills. An example quotation for developing a skill in singing was:

“*Yeah, I had a go at singing with my guitar, and I enjoyed that because that was entertaining, and I do sound like I've improved.”* (other Expert by Experience 2021 E).

#### 3.4.5 Re-evaluation of life because the world will change

Most Experts by Experience made lasting changes to their lives during and immediately following the first UK lockdown. One other Expert by Experience did not. Most also continued to believe that the COVID-19 pandemic would have a long-term effect on their lives nearing the end of lockdown three, even if they did not catch it. However, dissent was evident from a person physically disabled, using a wheelchair:

“*Well before today funnily enough I would have said yes, but I've been out in a park today for the first time since lockdown, and it felt quite natural. So as of a few minutes ago I'm now going to say no I don't think so.”* (other Expert by Experience 2021 K).

Changes, toward the end of lockdown one in 2020, by frequency included: planning ahead, increasing important relationships, becoming less materialistic, traveling more outside UK, buying a motorbike or car, seizing the day, shopping more locally through walking, using a car less, keeping garden tidy, doing more exercise, returning to Slimming World, doing more writing, upskilling in maths and IT, increasing lobbying and thanking others, working more from home, spending more time face to face, being careful about touching things, increasing event participations, moving house. Planning was apparent more often by Experts by Experience in 2020 compared with 2021. Several Experts by Experience moved home, partly explaining this reduction.

In 2021, there were also more defined long-term goals of all Experts by Experience, for example, regaining one's voice to make choices, maintaining fear of strangers socially, maintaining social distancing, creating a better diet, contacting people more often, undertaking more me/personal care time.

Between the two UK lockdowns in 2020 and 2021 there was a notion of slowing down for all Experts by Experience. Their aim was to improve mental health, and by learning to know themselves more, for example:

“*Yes, I do feel my mental health changed, and I feel that it's changed for the better, which is probably quite different to most people… I'm not rushing around and going here, there and everywhere, I've had the time to learn how to pace myself* .” (other Expert by Experience 2021 O).

“*I might choose a slightly less busy life, I might. Knowing myself yeah, hard to politely disentangle.”* (person with earlier mental distress 2021 P).

## 4 Discussion

### 4.1 Importance of critical public health theory

The COVID-19 pandemic or syndemic was the most indiscriminating in recent human history leading to high rates of mortality in vulnerable people across the world, especially people who were disabled/CEV/chronic illnesses/shielding (1, 11, 129). Given that the International Pandemic Preparedness Secretariat activity will result in the existence of globally available medicine within the first 100 days of a future pandemic, and that the World Health Organization Zero Treaty acceptance strengthens pandemic prevention, preparedness and response, there remains huge challenges of immediate emergency actions to safeguard the lives of citizens (108). In this study Experts by Experience who self-identified with pre-COVID-19 mental distress were compared with others who had physical impairments of mobility, sight, those CEV/chronic illnesses and their carers, including intellectual disabilities. Those with hidden disabilities self-identified either with pre-COVID mental distress or had visual challenges. Below is discussed the importance of critical public health theory for four of the five pillars which characterize those people who volunteered their time for this study to save lives of vulnerable or bioprecarious people with disabilities/CEV/chronic illnesses/shielding for 100 days, and beyond in case of future emergencies (3, 10, 13).

### 4.2 Pillar one—legacy

#### 4.2.1 Use of social media vs. the digital divide

Experts by Experience grew in confidence using social media as COVID-19 lockdowns proceeded unlike others' reports (74, 75). Views on social media by Experts by Experience in this study were equally proportioned, irrespective of disability. When positive, benefits were through making new contacts, for mutual support, and the advantages of online communication in agreement with earlier reports (78, 98). When negative, disadvantages were through destructive forces of misinformation, tiring sitting at a screen, missing clues about people when not engaging in person/in venue in agreement with other earlier reports (76, 77).

#### 4.2.2 Coping strategies

Individual Experts by Experience mentioned more than 30 coping strategies to fill their lockdown time, although not everybody enjoyed keeping busy, in contrast to an earlier report (94). People with earlier mental distress undertook less exercise during the first lockdown, in agreement with others (47–50). However, any differences between people with earlier mental distress, and other Experts by Experience disappeared near the end of the third UK lockdown. People with earlier mental distress were likely to have already understood the importance of developing coping strategies during recovery training following ill-health; most were able to adapt their coping strategies during lockdowns by listing a huge variety of activities, unlike early research findings (92). During this study, individual other Experts by Experience did more cleaning, and housework. Earlier reports have not made such detailed comparisons (46, 92). However, storytelling might provide a wide range of reported self-initiated coping strategies (63, 64).

#### 4.2.3 Developing new skills

Individual other Experts by Experience developed 19 new skills between the first and third UK lockdowns. Notably, other Experts by Experience learnt via online social media platforms and, studied to pass qualifications to aid their careers. People with earlier mental distress also developed new skills, including indoor exercising. Such a finding is under-reported in the literature.

#### 4.2.4 Re-evaluating of life because the world will change

Individual lives of Experts by Experience were re-evaluated and most thought there would be long-term effects of COVID-19. However, across both lockdowns, a quarter of the Experts by Experience from the current study believed COVID-19 would have no effect on their lives. Of the 29 distinct changes suggested, most mentioned were the importance of relationships, becoming less materialistic, more traveling outside the UK, buying a motorbike or car, slowing down, working from home more, and moving home. Earlier studies also mentioned the importance of relationships (46, 72, 92, 95).

#### 4.2.5 Celebrating lockdown' ends

Toward the end of both lockdowns most individual Experts by Experience voiced planned celebrations in their communities, holding positive outlooks, especially looking forward to resuming their work at/income from universities. However, a significant proportion did not plan to celebrate due to insufficient funds in agreement with the findings of Vaitsiakhovich et al. (101). Other Experts by Experience hoped to visit coffee shops, have meals out, and attend family gatherings, more at the end of the first UK lockdown. Any differences between Experts by Experience had disappeared by the end of the third lockdown, a year later, with more than ten different types of celebration planned by all.

### 4.3 Pillar two—health inequalities result from underlying social arrangements or institutions

#### 4.3.1 Activism

Certainly, community face-to-face volunteering by a sub-set of Experts by Experience was less during the COVID-19 pandemic. Yet people visually challenged, or living following stroke, increased their community telephone support during this study. Such a possibility of beneficial advocacy using lay-people was earlier suggested (99).

#### 4.3.2 Loneliness/isolation

In this study, individual challenges of other Experts by Experience were increased loneliness when living alone, but not people living alone with earlier mental distress. This contrasts with earlier findings (81, 83–85, 93). It was people visually challenged, living alone who were lonely. Yet, Experts by Experience with earlier mental distress, resulting in hospitalizations, equated their ability to cope with living alone, and therefore lockdowns; these may be new findings. Less than half of the Experts by Experience considered themselves isolated, during both lockdowns. This may be due to these Experts by Experience associates being a cohesive IMPACT group, communicating with each other through online meetings during lockdowns (excepting people who were visually challenged) in agreement with earlier findings (98, 130).

### 4.4 Pillar three—over-medicalization and the dominance of medical frames of reference

While there is dominance of medical frames of reference, most Experts by Experience shielded much longer than the 12 weeks recommended by UK Government letter. Many were also shielding a year later and none caught COVID-19 during this study. Therefore, the questioning of success of lockdowns and shielding during the UK COVID-19 Inquiry^5^ when there was increasing widespread UK vaccination by the conclusion of this study, demonstrated a public health success for these Experts by Experience. However, during the period of this study Experts by Experience were unable to newly access health, social care, and had respite challenges in agreement with others (51, 52). No rapid support was available to any Experts by Experience despite being CEV/chronic illnesses in agreement with others (53, 54). Initially, shielding affected some Experts by Experience with earlier mental distress, so demonstrating perpetuation of existing mental health inequalities, and social injustice as reported by others (55–58). This contrasts with findings that there were facilitators in the review of Croft and Fraser (42).

### 4.5 Pillar four—commitment to health equity

#### 4.5.1 Government controlled entitlements

Socioeconomic challenges were known to this IMPACT group because all lost most income streams, occupation, and therefore quality of life from their involvement in student selection, teaching and learning within this, and other universities because of their 0 h contracts and ineligibility for any UK Government grants, as was evident overseas (41, 70). This concurs with earlier reports of an ableist powerful majority considering their own needs foremost (40, 41, 69, 71).

#### 4.5.2 Diminished quality of life

Environmental challenges reduced quality of life, including, for example, accessibility of shops to buy food, GP surgeries/mental health/other health services/medication, COVID-19 vaccinations, social services support and respite care, transportation/buses, and public buildings for financial support/disability benefits, and employment support agreeing with earlier reports (45, 69, 92, 96). However, in this study following a year of hardship Experts by Experience normalized their reduced quality of life, being thankful they remained alive, especially people with mobility challenges, in contrast to an earlier report (86).

#### 4.5.3 People with hidden disabilities

Environmental and community challenges occurred for people with hidden disabilities, for example, people with visual challenges, and people with earlier mental/psychosocial distress. Citizens publicly abused people with hidden disabilities, for example, seeking food, medicines/medical support, and undertaking necessary travel during lockdowns. Citizens particularly abused people visually challenged (without a guide dog, or stick) possibly because of difficulties reading any written instructions to navigate shops/supermarkets during social distancing requirements. Therefore, people visually challenged did not leave their homes, with consequential reduced mobility, in agreement with an earlier report (46). Next affected were people with hidden, earlier mental distress during the first UK lockdown in 2020, in agreement with others (92, 102).

#### 4.5.4 Loss of stability in outlook

Individual loss of stability in outlook was greatest as lockdown one proceeded, especially people with earlier mental distress, in agreement early reports (92, 131). A year later, while all Experts by Experience voiced frustration and anger, they believe loss of stability in outlook had reduced, irrespective of whether they had earlier mental distress or not, in contrast with findings 3 months following the first lockdown for people with earlier mental distress and others (58, 73). This may be due to greater resilience through experiencing earlier trauma and hardship. Experts by Experience who were visually challenged reported their mental health had declined during both lockdowns, in agreement for increased anxiety, during the first lockdown (46).

### 4.6 Dynamic continuum identified

Character strengths/positive psychology of all Experts by Experience associates dynamically varied between interviews, a year apart, especially with respect to loss of stability in outlook. Thus, responses were identified as a continuum also relying on the findings of others (9, 91, 100). Trends due to hidden, mental/psychosocial and physical disabilities were found that both agreed and disagreed with earlier reports (31, 32) Agreement occurred for differentiation in need of provision for those Experts by Experience visually challenged and/or mental/psychosocially distressed, as earlier reported through review, although there was little evidence of any facilitators (42–44). Coincidentally, these people had hidden disabilities. As a cohesive group Experts by Experience were more positive overall, which may have contributed to them surviving the COVID-19 pandemic.

### 4.7 Limitations, strengths and insights

Experts by Experience associates within this UK university (an IMPACT group) contained people with disabilities, CEV/chronic illness, and carers (*n* = 23). They experienced a wide range of challenges during the COVID-19 pandemic. Critical public health theory of inequalities and equities in health used to derive the themes in this study were lack of income and occupation rather than any educational considerations. A social model of disability was not exclusively used in this study. Further analysis of this data and that of others might distinguish whether results and their interpretations might change. It was not possible to distinguish intersectional challenges during this study due to small sample size. In addition, no demographic information was collected because all Experts by Experience lived in one region of UK, and there was a risk of identification. The University ethics committee approved all studies, and studies used well established planning, interview methodology, recording, transcription, and qualitative thematic analyses with coding, and internal fidelity checking techniques. Experts by Experience were known to the researcher for up to 13 years through earlier involvement, conducting interviews during other research projects, and the researcher also attended pre-COVID-19 face-to-face IMPACT group meetings at this university. The Experts by Experience interviewed acknowledged this researcher's earlier career in academic scientific research, and publishing. Therefore, this researcher's dual role—also as an Expert by Experience—enabled interview participants to feel comfortable sharing their views widely as each question was asked. Because there was professional structure to the interviews, and the researcher did not over-reach boundaries, potential bias was eliminated. Data interpretation was completed anonymously on four independent occasions. All four analyses were compared, and contrasted before generative themes were finalized, again to reduce bias. Experts by Experience, and researcher contrasted with other UK university, well-funded teams, undertaking research using large-scale external surveys, interviewing financially maintained panels of selected/self-selected people that met predefined criteria. In addition, there may be significant differences in voices of people who were renumerated for their time as Experts by Experience, compared with this IMPACT group of volunteer/Experts by Experience associates of a university who wished for this research. The latter suggested this study for the benefit of other people with disabilities, CEV/chronic illnesses, and carers. They wished to provide suggestions to alleviate a future pandemic that would benefit vulnerable or bioprecarious people this IMPACT group identified with. The cohesive Experts by Experience associates within a UK university (IMPACT) had individual personal insights to report lived experiences during the COVID-19 pandemic. However, findings reflect the specific experiences of these Experts by Experience and might not represent all people with disabilities in the UK.

### 4.8 Recommendations to reduce mortality during future pandemics

#### 4.8.1 To combat initial spread

(1) **Recognize** the World Health Organization agreed Pandemic Accord/Instrument pandemic prevention, preparedness, and response (PPPR), and the World Health Organization International Health Regulations (IHR).
(2) **Enact** new/temporality appeal legislation by national governments following point (1).
(3) **Enact** new legislation, containing penalties, to reduce other citizens abusing people with disabilities, especially people with hidden disabilities, and people with CEV/chronic illnesses, by an ableist majority.
(4) **Educate** (including annual re-examination/certification) government agents planning for pandemics, disaster relief personnel, people employed in health and social care, in the needs and risk to vulnerable groups of people, including people with all types of mental/psychosocial and physical disabilities and people with CEV/chronic illnesses.

#### 4.8.2 To combat mortality of vulnerable or bioprecarious citizens

(5) **New funding** for increased needs of vulnerable groups, including people with hidden, and other disabilities, people with CEV/chronic illnesses and, their carers. This will include communications for emergencies, and other guidelines to keep vulnerable citizens alive, increase health and social care needs (including unmet mental health need/support for people with hidden disabilities, GP appointments, respite for carers), access to food, medicines, testing, vaccinations, transportation, employment protection/adjustments for home working/information for employers.
(6) **Consult**, prior/early with stakeholders/people with disabilities/CEV/chronic illnesses who experience health and social care, socio-economic, community, environmental, intersectional, and individual challenges, including carers by government agencies to inform point (7).
(7) **Communicate** immediate, effective, consistent emergency actions people need to take, and their priority entitlements to remain alive in multiple accessible formats, and methods by government agents.
(8) **Proactively** contact people with hidden disabilities, especially people visually challenged, inviting them for early mental health/psychological support by national health and social care services.
(9) **Proactively** contact people with CEV/chronic illnesses, inviting them for new, early mental health/psychological support, including maintenance of such support for people with earlier mental/psychosocial distress by national health and social care services.
(10) **Create** a single list of people with disabilities, people CEV/chronic illness according to detailed, medical diagnoses of health challenges, and people vulnerable according to their complex socio-economic indicators through environment, community, individual and intersectionality (gender, ethnicity, class, geography; obtainable from UK Office of National Statistics, or any nation's equivalent), and individual needs by government agents.
(11) **Choose** contact method preferences of people with disabilities, CEV/chronic illnesses/shielding to rapidly disseminate emergency information, and guidelines from stored list by a nation's health and social care systems.
(12) **Maintain**, and update single list by government agents/people employed across any nation's health, and social care organizations.
(13) **Store** single list revised/reformatted with evolving technology for rapid data sharing across organizations by government agents/people employed across any nation's health, and social care organizations.
(14) **Provide** further information to aid survival during any future lockdowns to people on stored list, for example, different lists of possible coping strategies, with links to online “how to” information, based on people with diverse disabilities, especially people with hidden disabilities, and people with CEV/chronic illnesses; how to develop new skills, and join online certificated training courses to enter new careers; how to reflect, and develop individual, and families' relationships over their life courses in light of the current emergency, and ideas for planning low costs end of lockdown celebrations, giving hope for a better future.
(15) **Create** accessible directory of third sector organizations, through providing new funding for National Council for Voluntary Organizations (NVCO), or equivalents across other nations, and regularly maintain/update to include national, and local organizations, classified by alleviation of need offered/availability, and county/community, including contact information for example, email addresses, telephone helplines, and online resources.
(16) **Make available** to all directory at (15), including national organizations, providers, and the third sector, to provide effective support to vulnerable groups of people/individuals, also through signposting by advocates.
(17) **Combat** loneliness to access communities, and groups of people using social media platforms through using directory at (15).
(18) **Engage** all stakeholders to regularly practice, monitor, review and update 1–18 to ensure validity.

## 5 Conclusions

Experts by Experiences associates within a UK university were service users, with a wide range of disabilities, and their carers, most of whom were also CEV during the COVID-19 pandemic or syndemic. Their insights interpreted through a lens of critical public health of inequality and inequity in health to include socioeconomic complexities of environmental, community, and individual challenges, suggested that this vulnerable, or precarious group, needed differential prioritization by nations' governments to remain alive, especially those with hidden disabilities. Character strengths/positive psychology responses were complex, and dynamically changing as the COVID-19 pandemic progressed. Vulnerable people with disabilities, their carers, allies and people CEV/chronic illness deserve to remain alive, and experience equitable lives of quality they self-determine. This study provided recommendations to combat initial spread, mortality and inequity to prepare for future pandemics. Preparedness requires ensuring the functioning of all systems despite circumstances. Long-term research and monitoring might prepare for various health threats, which will enable anticipation, rapid response, support for decision-making and prevention, including all stakeholder involvement. International cooperation in preparedness is important.

## Data Availability

The raw data supporting the conclusions of this article will be made available by the authors, without undue reservation.

## Footnotes

^1^Disability Rights UK www.disabilityrightsuk.org.

^2^Comensus www.uclan.ac.uk.

^3^Shaping Our Lives www.shapingourlives.org.uk.

^4^Survivor Users Network www.nsun.org.uk.

^5^UK COVID-19 Public Inquiry https://COVID19.public-inquiry.uk/.

## References

[1] Caron RM Adegboye ARA. COVID-19: A syndemic requiring an integrated approach for marginalized populations. Front Public Health. (2021) 9:675280. 10.3389/fpubh.2021.675280 34046392 PMC8144466

[2] Liu E Dean CA Elder KT. Editorial: The impact of COVID-19 on vulnerable populations. Front Public Health. (2023) 11:1267723. 10.3389/fpubh.2023.1267723 37744477 PMC10515203

[3] Ramírez SJ. Bioprecariousness and vulnerability: An ethical approach to the issues of access to public health. Ethic Med Public Health. (2025) 33:101066. 10.1016/j.jemep.2025.101066

[4] Yang QE Xiaodan Ma X Zeng L Wang Q Li M Teng L. Interphylum dissemination of NDM-5-positive plasmids in hospital wastewater from Fuzhou, China: A single centre, culture-independent, plasmid transmission study. Lancet Microbe. (2024) 5:e12–23. 10.1016/S2666-5247(23)00227-6 38006896

[5] British Medical Association (BMA). The Public Health Response by UK Governments to COVID-19 (2024). Available online at: https://www.bma.org.uk/media/5980/bma-covid-review-report-4-28-july-2022.pdf (Accessed June 11, 2025).

[6] British Medical Association (BMA). The Impact of the Pandemic on Population Health and Health Inequalities (2024). Available online at: https://www.bma.org.uk/media/bzxla0fv/bma-covid-review-report-5-september-2024.pdf (Accessed June 11, 2025).

[7] Mitra M Turk MA. People with disabilities, community living, and COVID-19. Disabil Health J. (2022) 15:101230. 10.1016/j.dhjo.2021.101230 34906357 PMC8665669

[8] Rooney JM Brain P. ‘Psychosocial health education within community mental health inpatient recovery wards in UK: patients' voices.' *J Educ Train Stud*. (2024) 12:20–34. 10.11114/jets.v12i1.645339100657

[9] Tichy NG Hergenrather KC Dos Santos B McGuire-Kuletz M Beland L. The impact of COVID-19 on persons with disabilities: a systematic review of literature. Rehab Counsel Educ J. (2022) 11. 10.52017/001c.56915

[10] World Health Organization. International Classification of Functioning, Disability and Health. Geneva: World Health Organization (2025).

[11] Bosworth ML Ayoubkhani D Nafilyan V Foubert J Glickman M Davey C. Deaths involving COVID-19 by self-reported disability status during the first two waves of the COVID-19 pandemic in England: a retrospective, population-based cohort study. Lancet Public Health. (2021) 6:e817–25. 10.1016/S2468-2667(21)00206-1 34626547 PMC8494502

[12] Office for National Statistics UK (ONS). Updated Estimates of Coronavirus (COVID-19) Related Deaths by Disability Status, England: 24 January 2020 to 9 March 2022 (2022a). Available online at: https://www.ons.GOV.UK/peoplepopulationandcommunity/birthsdeathsandmarriages/deaths/articles/coronavirusCOVID19relateddeathsbydisabilitystatusenglandandwales/24january2020to9march2022 (Accessed June 11, 2025).

[13] Schrecker T. What is critical about critical public health? Focus on health inequalities. Crit Public Health. (2021) 32:139–44. 10.1080/09581596.2021.1905776

[14] Chen DT Wang Y-J. Inequality-related health and social factors and their impact on well-being during the COVID-19 pandemic: findings from a national survey in the UK. Int J Environ Res Public Health. (2021) 18:1014. 10.3390/ijerph18031014 33498842 PMC7908210

[15] Hanson S Belderson P Ward E Naughton F Notley C. Lest we forget. Illuminating lived experience of the COVID-19 pandemic and lockdown. Soc Sci Med. (2023) 332:116080. 10.1016/j.socscimed.2023.116080 37451941

[16] GOV.UK. Influenza Preparedness Strategy (2011). Available online at: https://assets.publishing.service.GOV.UK/media/5a7c4767e5274a2041cf2ee3/dh_131040.pdf (Accessed June 11, 2025).

[17] GOV.UK. Exercise Cygnus (2017). Available online at: https://assets.publishing.service.GOV.UK/media/5f8eb911d3bf7f49a1ce842c/exercise-cygnus-report.pdf (Accessed June 11, 2025).

[18] GOV.UK. Policy Paper on Preparedness (2020). Available online at: https://www.GOV.UK/government/publications/uk-pandemic-preparedness/uk-pandemic-preparedness (Accessed June 11, 2025).

[19] Rietveld J Hobson T Mani L Avin S Sundaram L. The UK's pandemic preparedness and early response to the COVID-19 pandemic. Glob Public Health. (2024) 19:2415499. 10.1080/17441692.2024.2415499 39432455

[20] Walby S. The COVID pandemic and social theory: social democracy and public health in the crisis. Eur J Soc Theory. (2021) 24:22–43. 10.1177/1368431020970127

[21] Radburn M Stott C Bryant R Morgan B Tallent D Davidson L. Group processes and interoperability: a longitudinal case study analysis of the UK's civil contingency response to COVID-19. J Conting Crisis Manag. (2023) 31:121–33. 10.1111/1468-5973.12424 40479403 PMC9350367

[22] Bromfield N Carey G. What can be learned at the intersection of crisis management and administrative burdens? Evidence from a systematic review of the governance of Australia's national disability insurance scheme during COVID-19. Soc Pol Adm. (2025) 1–12. 10.1111/spol.13136

[23] Wang Y Campos-Carrau M. Civic engagement and health communication in the context of COVID-19 crisis: A systematic review (Compromiso cívico y comunicación sanitaria en el contexto de la crisis del COVID-19: una revisión sistemática). Eur Pub Soc Innov Rev. (2024) 9:1–22. Spanish. 10.31637/epsir-2024-796

[24] Rooney JM Foxall J Parkinson K Harris E Collins H Mariga P. An evaluation of the Health Equalities Framework for people with a learning disability. Learn Disabil Pract. (2018) 21:32–7. 10.7748/ldp.2018.e1886

[25] Digital NHS. Tracking Healthcare Activity and Outcomes for Shielded Patients, England (2020). Available online at: https://digital.nhs.uk/data-and-information/publications/statistical/mi-tracking-healthcare-activity-and-outcomes-for-shielded-patients-england/latest/content (Accessed June 11, 2025).

[26] World Health Organization. Disability. Geneva: World Health Organization (2024).

[27] Beresford P Farr M Hickey G Kaur M Ocloo J Tembo D. COVID-19 and Co-Production in Health and Social Care Research, Policy and Practice: Volume 1: The Challenges and Necessity of Co-Production. Bristol: Policy Press, Bristol University Press (2021).

[28] Duffy J Cameron C Casey H Beresford P McLaughlin H. Service user involvement and COVID-19-an afterthought? Brit. J Soc Work. (2022) 52:2384–402. 10.1093/bjsw/bcac007

[29] Lugo-Agudelo LH Spir Brunal MA Posada Borrero AM Cruz Sarmiento KM Velasquez Correa JC Di Dio Castagna Iannini R. Countries response for people with disabilities during the COVID-19 pandemic. Front Rehabil Sci. (2022) 2:796074. 10.3389/fresc.2021.796074 36188782 PMC9397944

[30] Snooks H Watkins A Lyons J Akbari A Bailey R Bethell L. Did the UK's public health shielding policy protect the clinically extremely vulnerable during the COVID-19 pandemic in Wales? Results of EVITE immunity, a linked data retrospective study. Public Health. (2023) 218:12–20. 10.1016/j.puhe.2023.02.008 36933354 PMC9928733

[31] Hendry G Wilson C Orr M Scullion R. “I just stay in the house so I don't need to explain”: A qualitative investigation of persons with invisible disabilities *Disabil*. (2022) 2:145–63. 10.3390/disabilities2010012

[32] Reynolds GS. “Other people thought i was dirty or crazy”: identity conversations about medical exceptions to masking. Commun Stud. (2024) 75:689–711. 10.1080/10510974.2024.2390270

[33] Beevi SS Verma VK Chowdary R. Challenges and resilience of disabled people during COVID-19. In:G. Bennett and E. Goodall, editors. The Palgrave Encyclopedia of Disability. London: Palgrave Macmillan, Cham.

[34] Abrams T Abbott D. Disability, deadly discourse, and collectivity amid Coronavirus (COVID-19). Scand J Disabil Res. (2020) 22:168–74. 10.16993/sjdr.732

[35] Dilodovico A. Disability studies. The Year's Work in Critical and Cultural Theory. (2023) 31:61–75. 10.1093/ywcct/mbad014

[36] Gummesson K Forsell K Johansson S Gustavsson C. How did people with impairments perceive public information during the COVID-19 pandemic and what are their suggestions for accessible crisis information? Scand J Disabil Res. (2024) 26:601–19. 10.16993/sjdr.1129

[37] Li L Taeihagh A Tan SY. A scoping review of the impacts of COVID-19 physical distancing measures on vulnerable population groups. Nat Commun. (2023) 14:599. 10.1038/s41467-023-36267-9 36737447 PMC9897623

[38] Goggin G Ellis K. Disability, communication, and life itself in the COVID-19 pandemic. Health Sociol Rev. (2020) 29:168–76. 10.1080/14461242.2020.1784020 33411654

[39] Shakespeare T. Triple jeopardy: disabled people and the COVID-19 pandemic. Lancet. (2021) 397:1331–3. 10.1016/S0140-6736(21)00625-5 33740474 PMC7963443

[40] Read S Parfitt A Bush T Simmons B Levinson M. Disabled People's experiences of the coronavirus pandemic: a call to action for social change. Soc Incl. (2023) 11:38–47. 10.17645/si.v11i1.5721

[41] Taher A Power E Payne G. ‘You're just invisible and you don't matter at all': the structural violence of the COVID-19 Canada Emergency Response Benefit (CERB). J Crit Public Health. (2025) 2:23–39. 10.55016/ojs/jcph.v2i1.77909

[42] Croft S Fraser S. A scoping review of barriers and facilitators affecting the lives of people with disabilities during COVID-19. Front Rehabil Sci. (2022) 2:784450. 10.3389/fresc.2021.784450 36188856 PMC9397712

[43] Ganesh GS Goyal C. Impact of COVID-19 on persons with disabilities. In:G. Bennett E. Goodall, editors. The Palgrave Encyclopedia of Disability. London: Palgrave Macmillan, Cham (2025).

[44] Lombe W. A synthesis: exploring the interconnection between disability and COVID-19. J Prevent Rehab Med. (2023) 5:37–43. 10.21617/jprm20232

[45] Holm ME Sainio P Suvisaari J Sääksjärvi K Jääskeläinen T Parikka S. Differences in unfavourable lifestyle changes during the COVID-19 pandemic between people with and without disabilities in Finland: psychological distress as a mediator. Int J Environ Res Public Health. (2022) 19:6971–86. 10.3390/ijerph19126971 35742223 PMC9223132

[46] Halpern N Rickly JM Hansen M Fellenor J. COVID-19 and vision impairment: constraints, negotiation, participation, and well-being during lockdown in The United Kingdom. Br J Vis Impair. (2023) 41:3–19. 10.1177/02646196211009931 36605535 PMC9791003

[47] Faulkner J O'Brien WJ McGrane B Wadsworth D Batten J Askewe CD. Physical activity, mental health and well-being of adults during initial COVID-19 containment strategies: a multi-country cross-sectional analysis. J Sci Med Sport. (2021) 24:320–26. 10.1016/j.jsams.2020.11.016 33341382 PMC7711171

[48] Jacob L Tully MA Barnett Y Lopez-Sanchez GF Butler L Schuch FR. The relationship between physical activity and mental health in a sample of the UK public: a cross-sectional study during the implementation of COVID-19 social distancing measures. Ment Health Phys Act. (2020) 19:100345. 10.1016/j.mhpa.2020.100345 32834833 PMC7378001

[49] López-Sánchez GF López-Bueno R Gil- Salmerón A Zauder R Skalska M Jastrzebska J. Comparison of physical activity levels in Spanish adults with chronic conditions before and during COVID-19 quarantine. Eur J Public Health. (2020) 31:161–6. 10.1093/eurpub/ckaa159 32761181 PMC7454536

[50] Violant-Holz V Gallego-Jiménez GM González-González CS Muñoz-Violant S Rodriguez MJ Sansano-Nadal O. Psychological health and physical activity levels during the COVID-19 pandemic: a systematic review. Int J Environ Res Public Health. (2020) 17:9419. 10.3390/ijerph17249419 33334073 PMC7765528

[51] McBride-Henry K Nazari Orakani S Good G Roguski M Officer TN. Disabled people's experiences accessing healthcare services during the COVID-19 pandemic: A scoping review. BMC Health Serv Res. (2023) 23:346–84. 10.1186/s12913-023-09336-4 37024832 PMC10078067

[52] Ndlovu L Mudonhi N Sibanda N Nunu WN Manyeruke N. Effects of COVID-19 among people with disabilities in Plumtree, Zimbabwe: a mixed-method Survey. Health Serv Insights. (2024) 17:1–17. 10.1177/11786329241274484 39193549 PMC11348347

[53] Bramanti SM Trumello C Lombardi L Babore A. COVID-19 and chronic disease patients: Perceived stress, worry, and emotional regulation strategies. Rehab Psychol. (2021) 66:380–5. 10.1037/rep0000409 34591525

[54] Cox N Raizada SR Barkham N Venkatachalam S Sheeran TP Adizie T. The impact of the COVID-19 pandemic and stringent social distancing measures on health-related quality of life and COVID-19 infection rates in patients with rheumatic disease: A longitudinal analysis through the pandemic. Rheumatol Adv Pract. (2023) 7:rkad009. 10.1093/rap/rkad009 36751643 PMC9897299

[55] Manning RB Cipollina R Lowe SR Bogart KR Ostrove JM Adler JM. Barriers to mental health service use among people with disabilities during the COVID-19 pandemic. Rehab. Psychol. (2023) 68:351–61. 10.1037/rep0000512 37470994 PMC10799191

[56] Morris SG Kudrna L Martin J. Mental health and life satisfaction among those advised to shield during the COVID-19 pandemic in the UK: A secondary analysis of the understanding society longitudinal study. Front Public Health. (2023) 11:1235903. 10.3389/fpubh.2023.1235903 37829093 PMC10566375

[57] Newbronner E Walker L Wadman R Crosland S Johnston G Heron P. Influences on the physical and mental health of people with serious mental ill-health during the COVID-19 pandemic: a qualitative interview study, Int. J Q Stud Health Well-being. (2022) 17:2122135. 10.1080/17482631.2022.2122135 36073745 PMC9467576

[58] Shah P Hardy J Birken M Foye U Rowan-Olive R Nyikavaranda P. What has changed in the experience of people with mental health problems during the COVID-19 pandemic: a coproduced, qualitative interview study. Soc Psych Psych Epid. (2022) 57:1291–303. 10.1007/s00127-022-02254-6 35267053 PMC8908744

[59] World Health Organization. Preparedness and resilience for emerging threats. Module 1: planning for respiratory pathogen pandemics. Geneva: World Health Organization (2024d).

[60] Shikako K Lencucha R Hunt M Jodoin S Elsabbagh M Hudson M. How did governments address the needs of people with disabilities during the COVID-19 pandemic? An analysis of 14 countries' policies based on the UN Convention on the Rights of Persons with Disabilities. Int J Health Policy Manag. (2023) 12:1–12. 10.34172/ijhpm.2023.7111 37579394 PMC10425656

[61] Liu H Coveney C. Public participation in the time of COVID-19: response from the international disability movement. Soc Incl. (2025) 13:124–35. 10.17645/si.910535184367

[62] Wolbring G Lillywhite A. Coverage of allies, allyship and disabled people: a scoping review. Societies (2023) 13:241. 10.3390/soc13110241

[63] Centre for Mental Health. A Year in Our Lives: An Anthology 2022 (2022). Available online at: http://www.centreformentalhealth.org.uk/publications/year-our-lives-anthology (Accessed June 11, 2025).

[64] Gilbert D. Reaching Inland (2022). Available online at: http://www.centreformentalhealth.org.uk//publications/reaching-inland (Accessed June 11, 2025).

[65] Berger ZD Evans NG Phelan AL Silverman RD. COVID-19: control measures must be equable and inclusive. BMJ. (2020) 368:m1141. 10.1136/bmj.m1141 32198146

[66] Cullingworth J Watson N Shakespeare T Brunner R Pearson C Scherer N. “They have been a saving grace in all this”: the role of the third sector in disabled people's experiences of COVID-19 and implications for sector–state relations. Volunt Sect Rev. (2024) 15:92–109. 10.1332/204080521X16593450428164

[67] Niles CA Hande MJ Schormans AF Porch W Mahipaul S Anderson J. What and who are “essential”? A disability justice perspective on COVID-19 measures and the diverse disability communities in Ontario. Stud Soc Justice. (2025) 19:104–29. 10.26522/ssj.v19i1.4099

[68] Whitton A Callis L Bednar H Ellis E Skaggs D Hinds C. Partnering with disability-led organizations to prepare and respond to public health emergencies. J Emerg Manag. (2025) 23:191–9. 10.5055/jem.0878 40186469

[69] Epstein S Campanile J Cerilli C Gajwani P Varadaraj V Swenor BK. New obstacles and widening gaps: a qualitative study of the effects of the COVID-19 pandemic on U.S. adults with disabilities. Disabil Health J. (2021) 14:101103. 10.1016/j.dhjo.2021.101103 33840617 PMC9760300

[70] Kerschbaumer L Crossett L Holaus M Costa U. COVID-19 and health inequalities: the impact of social determinants of health on individuals affected by poverty. Health Policy Technol. (2024) 13:100803. 10.1016/j.hlpt.2023.100803

[71] Mladenov T Brennan CS. The global COVID-19 disability rights monitor: implementation, findings, disability studies. Disabil Soc. (2021) 36:1356–61. 10.1080/09687599.2021.1920371

[72] Pearson C Watson N Brunner R Cullingworth J Hameed S Scherer N. COVID-19 and the crisis in social care: exploring the experiences of disabled people in the pandemic. Soc Pol Soc. (2023) 22:515–30. 10.1017/S1474746422000112

[73] Finne E Nowak AC Razum O. Regional COVID-19 measures and effects on subjective well-being in Germany: observing trends over time with data from a large population survey. Front Public Health. (2025) 13:1523691. 10.3389/fpubh.2025.1523691 40084213 PMC11905229

[74] Cameron E Curran D. Digital risk and inequality. In:D. Curran, editor. Handbook on Risk and Inequality (Cheltenham, UK: Elgar Handbooks on Inequality, Edward Elgar Publishing) (2022). p. 88–105.

[75] Ellis K Kent M Locke K McRae L Dau D Peaty G. Smartphones, disability and the Australian experience of the COVID-19 pandemic for people who are blind and with low vision. Disabil Stud Q. (2021) 41. 10.18061/dsq.v41i3.8318

[76] Gao J Zheng P Jia Y Chen H Mao Y Chen S. Mental health problems and social media exposure during COVID-19 outbreak. PLoS ONE. (2020) 15:e0231924. 10.1371/journal.pone.0231924 32298385 PMC7162477

[77] Banerjee D Vaishnav M Rao TS Raju MSVK Dalal PK Javed A. Impact of the COVID-19 pandemic on psychosocial health and well-being in south-Asian (World Psychiatric Association Zone 16) countries: a systematic and advocacy review from The Indian Psychiatric Society. Indian J Psychiat. (2020) 62:S343–53. 10.4103/psychiatry.IndianJPsychiatry_1002_20 33227049 PMC7659771

[78] Dobransky K Hargittai E. Piercing the pandemic social bubble: disability and social media use about COVID-19. Am Behav Sci. (2021) 65:1698–720. 10.1177/00027642211003146 38603151 PMC8010377

[79] Björnsdóttir K. Lived experiences of people with intellectual disabilities during the COVID-19 pandemic from a crip time perspective. Canad J Disabil Stud. (2024) 13:73–104. 10.15353/cjds.v13i3.1162

[80] Bu F Steptoe A Fancourt D. Who is lonely in lockdown? Cross-cohort analyses on predictors of loneliness before and during the COVID-19 pandemic. Public Health. (2020) 186:31–4. 10.1016/j.puhe.2020.06.036 32768621 PMC7405905

[81] Groarke JM Berry E Graham-Wisener L McKenna-Plumley PE McGlinchey E Armour C. Loneliness in the UK during the COVID-19 pandemic: cross-sectional results from the COVID-19 psychological wellbeing study. PLoS ONE. (2020) 15:e0239698. 10.1371/journal.pone.0239698 32970764 PMC7513993

[82] Heinze N Castle CL Hussain SF Godier-McBard LR Kempapidis T Gomes RS. State anxiety in people living with disability and visual impairment during the COVID-19 pandemic. Disabilities. (2022) 2:235–46. 10.3390/disabilities2020017 PMC8458570 34568266

[83] Kavanagh A Hatton C Stancliffe RJ Aitken Z King T Hastings R. Health and healthcare for people with disabilities in the UK during the COVID-19 pandemic. Disabil Health J. (2021) 15:101171. 10.1016/j.dhjo.2021.101171 34330683 PMC8285926

[84] Lee B Rumrill P Mangadu T Estala-Gutierrez V Tansey TN Umucu E. The intermediary role of loneliness in the relationship between COVID-19 stress and maladaptive coping among people with disabilities and chronic health conditions. J Rehabil. (2021) 87:33–9. Available online at: https://link.gale.com/apps/doc/A661027846/HRCA?u=anon~508de7c0&sid=googleScholar&xid=b715d90d (Accessed June 11, 2025).

[85] Lewis K. COVID-19: preliminary data on the impact of social distancing on loneliness and mental health. J Psychiatr Pract. (2020) 26:400–4. 10.1097/PRA.0000000000000488 32936586

[86] Mittal K Philo Magdalene A Pathak D. Preventable losses: threatening rise in suicides during the pandemic. In:S. Pachauri and A. Pachauri, editors. Global Perspectives of COVID-19 Pandemic on Health, Education, and Role of Media (Singapore: Springer) (2023). p. 329–49.

[87] Neille J. I'm not ok, we are not ok': an exploration into the embodied precarity experienced by disabled people and their family members living in rural South Africa during the COVID-19 pandemic. Med Hum BMA. (2025) 0:1–10. 10.1136/medhum-2024-013026 39762027

[88] Tarsitani L Pinucci I Tedeschi F Patanè M Papola D Palantza C. Resilience of people with chronic medical conditions during the COVID-19 pandemic: a 1-year longitudinal prospective survey. BMC Psychiat. (2022) 22:633. 10.1186/s12888-022-04265-8 36183067 PMC9525930

[89] Umucu E Lee B. Examining the impact of COVID-19 on stress and coping strategies in individuals with disabilities and chronic conditions. Rehab Psychol. (2020) 65:193–8. 10.1037/rep0000328 32406739

[90] Eriksson M Nilsson EM Lundälv J. A scoping review of research exploring working life practices of people with disabilities during the COVID-19 Pandemic. Scand J Disabil Res. (2023) 25:241–55. 10.16993/sjdr.1012

[91] Fitzgerald S Chronister J Cabiles C Thomas T Zheng QM. The lived experience of people with disabilities during COVID-19. Rev Disabil Stud. (2024) 20. Available online at: https://rdsjournal.org/index.php/journal/article/view/1355 (Accessed June 11, 2025).

[92] Gillard S Dare C Hardy J Nyikaveranda P Rowan-Olive R Shah R. Experiences of living with mental health problems during the COVID-19 pandemic in the UK: a coproduced, participatory qualitative interview study. Soc Psych Psych Epid. (2021) 56:1447–57. 10.1007/s00127-021-02051-7 33665680 PMC7931976

[93] Holm ME Sainio P Parikka S Koskinen S. The effects of the COVID-19 pandemic on the psychosocial well-being of people with disabilities. Disabil Health J. (2022) 15:101224. 10.1016/j.dhjo.2021.101224 34690076 PMC8542066

[94] Lindsay S Ahmed H Apostolopoulos D. Facilitators for coping with the COVID-19 pandemic: online qualitative interviews comparing youth with and without disabilities. Disabil Health J. (2021) 14:101113. 10.1016/j.dhjo.2021.101113 34083178 PMC8436050

[95] Ramkissoon H. COVID-19 adaptive interventions: implications for wellbeing and quality-of-life. Front Psychol. (2022) 13:810951. 10.3389/fpsyg.2022.810951 35369239 PMC8968731

[96] Shakespeare T Watson N Brunner R Cullingworth J Hameed S Scherer N. Disabled people in Britain and the impact of the COVID-19 pandemic. Soc Pol Adm. (2021) 56:103–17. 10.1111/spol.12758 34548712 PMC8446989

[97] van der Velden PG Contino C de Vroege L Das M Bosmans M Zijlmans J. The prevalence of anxiety and depression symptoms (ADS), persistent and chronic ADS among the adult general population and specific subgroups before and during the COVID-19 pandemic until December 2021. J Affect Disord. (2023) 338:393–401. 10.1016/j.jad.2023.06.042 37364654 PMC10290740

[98] Wong J Yi PX Quek FYX Lua VYQ Majeed NM Hartanto A. A four-level meta-analytic review of the relationship between social media and well-being: A fresh perspective in the context of COVID-19. Curr Psychol. (2024) 43:14972–86. 10.1007/s12144-022-04092-w 36531193 PMC9748903

[99] Kahlon MK Aksan N Aubrey R Clark N Cowley-Morillo M Jacobs EA. Effect of layperson-delivered, empathy-focused program of telephone calls on loneliness, depression, and anxiety among adults during the COVID-19 pandemic a randomized clinical trial. JAMA Psychiat. (2021) 78:616–22. 10.1001/jamapsychiatry.2021.0113 33620417 PMC7903319

[100] Umucu E Lee B Genova HM Chopik WJ Sung C Yasuoka M. Character strengths across disabilities: an international exploratory study and implications for positive psychiatry and psychology. Front Psychiatr. (2022) 13:863977. 10.3389/fpsyt.2022.863977 35280155 PMC8914428

[101] Vaitsiakhovich N Landes SD Monnat SM. The role of perceived social support in subjective wellbeing among working-age U.S. adults with and without limitations in activities of daily living. Disabil Health J. (2025) 18:101705. 10.1016/j.dhjo.2024.101705 39242222 PMC11625594

[102] Healy J. The government hates disabled people. In:S. J. Macdonald and D. Peacock, editors. Handbook of Disability, Crime, and Justice. Oxford: Routledge (2025). p. 244–56.

[103] Lund EM Ayers KB. Ever-changing but always constant: “waves” of disability discrimination during the COVID-19 pandemic in the United States. Disabil Health J. (2022) 15:101374. 10.1016/j.dhjo.2022.101374 36156274 PMC9436863

[104] Okoro CA Strine TW McKnight-Eily L Verlenden J Hollis MD. Indicators of poor mental health and stressors during the COVID-19 pandemic, by disability status: a cross-sectional analysis. Disabil Health J. (2021) 14:101110. 10.1016/j.dhjo.2021.101110 33962896 PMC8436151

[105] Watson N Shakespeare T. Module 2 structural inequalities and disability, expert report for COVID-19 public inquiry (2023). Available online at: https://COVID19.public-inquiry.uk/documents/inq000280067-expert-report-titled-structural-inequalities-and-disability-by-professor-nick-watson-and-professor-tom-shakespeare-dated-21-september-2023/ (Accessed June 11, 2025).

[106] GOV.UK. Spring Statement 2025 Health and Disability Benefit Reforms (2025a). Available online at: https://www.GOV.UK/government/consultations/pathways-to-work-reforming-benefits-and-support-to-get-britain-working-green-paper/spring-statement-2025-health-and-disability-benefit-reforms-equality-analysis#policy-summary (Accessed June 11, 2025).

[107] World Health Organization. Statement on the Fifteenth Meeting of the IHR (2005) Emergency Committee on the COVID-19 Pandemic. Geneva: World Health Organization (2023).

[108] International Pandemic Preparedness Secretariat. 100 Days Mission Implementation Report-2023 (2024). Available online at: https://ippsecretariat.org/news/warnings-of-bare-rd-pipeline-for-top-pathogens-with-pandemic-potential-as-latest-100-days-mission-report-launched/ (Accessed June 11, 2025).

[109] British Broadcasting Company (BBC). WHO Agrees Legally Binding Pandemic Treaty (2025). Available online at: https://www.bbc.co.uk/news/articles/c7vn1r3ge2jo#:~:text=WHO%20agrees%20legally%20binding%20pandemic%20treatyandtext=Members%20of%20the%20World%20Health,during%20the%20COVID%2D19%20outbreak (Accessed June 11, 2025).

[110] Lazarus JV Martinez CP Kopka CJ Batista C El-Sadr WM Saenz R. Implications from COVID-19 for future pandemic global health governance. Clin Microb Infec. (2024) 35:576–81. 10.1016/j.cmi.2023.03.027 37011809 PMC10065870

[111] GOV.UK. Guidance for Local Resilience Forums. Identifying and supporting persons who are vulnerable in an emergency. Available online at: https://www.gov.uk/government/publications/identifying-people-who-are-vulnerable-in-a-crisis-guidance-for-emergency-planners-and-responders (Accessed June 11, 2025).

[112] GOV.UK. Accessible Online Advice for Disabled People (2025c). Available online at: https://prepare.campaign.GOV.UK/get-prepared-for-emergencies/advice-for-disabled-persons-and-carers/ (Accessed June 11, 2025).

[113] GOV.UK. Autumn Exercise announced (2025d). Available online at: https://www.GOV.UK/government/news/largest-ever-national-pandemic-response-exercise-to-strengthen-against-future-threats#:~:text=The%20national%20pandemic%20exercise%20is,event%20of%20another%20major%20pandemic (Accessed June 11, 2025)

[114] Office for National Statistics UK (ONS). Health Inequalities (2022b). Available online at: https://www.ons.GOV.UK/peoplepopulationandcommunity/healthandsocialcare/healthinequalities (Accessed June 11, 2025).

[115] McLaughlin H. What's in a name: ‘client', ‘patient', ‘customer', ‘consumer', ‘Expert by Experience', ‘service user'—what's next? Br J Soc Work. (2009) 39:1101–17. 10.1093/bjsw/bcm155

[116] International Federation of Social Workers (IFSW) and the International Association of Schools of Social Work (IASSW). Global Standards for the Education and Training of the Social Work Profession (2020). Available online at: https://www.iassw-aiets.org/wp-content/uploads/2018/08/Global-standards-for-the-education-and-training-of-the-social-work-profession.pdf (Accessed June 11, 2025).

[117] Nursing and Midwifery Council. The Code: Professional Standards of Practice and Behaviour for Nurses and Midwives. Standard 1:12 (2018a). London, UK: Nursing and Midwifery Council. Available online at: https://www.nmc.org.uk/standards/code/ (Accessed June 11, 2025).

[118] Nursing and Midwifery Council. Standards Framework for Nursing and Midwifery Education (2018b). Available online at: https://www.nmc.org.uk/standards/standards-for-nurses/ (Accessed June 11, 2025).

[119] Health and Care Professions Council. Standards of Education and Training Guidance. Standard 3.7 (2017). London, UK: Health and Care Professions Council. Available online at: https://www.hcpc-uk.org/resources/standards/standards-of-education-and-training/ (Accessed June 11, 2025).

[120] Royal College of Physicians. Faculty of Physician Associates (2020). Available online at: https://www.fparcp.co.uk/ (Accessed June 11, 2025)

[121] Braun V Clarke V. Using thematic analysis in psychology. Qual Res Psychol (2006) 3:77–101. 10.1191/1478088706qp063oa32100154

[122] Braun V Clarke V. Reflecting on reflexive thematic analysis. Qual Res Sport Exerc Health. (2019) 11:589–97. 10.1080/2159676X.2019.1628806

[123] Braun V Clarke V. To saturate or not to saturate? Questioning data saturation as a useful concept for thematic analysis and sample-size rationales. Qual Res Sport Exerc Health. (2021) 13:201–16. 10.1080/2159676X.2019.1704846

[124] Braun V Clarke V. Is thematic analysis used well in health psychology? A critical review of published research, with recommendations for quality practice and reporting. Health Psychol Rev. (2023) 17:695–718. 10.1080/17437199.2022.2161594 36656762

[125] Braun V Clarke V. Toward good practice in thematic analysis: avoiding common problems and be(com)ing a *knowing* researcher. Int J Transgend Health. (2023) 24:1–6. 10.1080/26895269.2022.2129597 36713144 PMC9879167

[126] Braun V Clarke V. Supporting best practice in reflexive thematic analysis reporting in Palliative Medicine: a review of published research and introduction to the Reflexive Thematic Analysis Reporting Guidelines (RTARG). Palliat Med. (2024) 38:608–16. 10.1177/02692163241234800 38469804 PMC11157981

[127] Braun V Clarke V. A critical review of the reporting of reflexive thematic analysis in Health Promotion International. Health Promot Int. (2024) 39:daae049. 10.1093/heapro/daae049 38805676 PMC11132294

[128] Clarke V Braun V Adams J Callaghan JE LaMarre A Semlyen J. “Being really confidently wrong”: Qualitative researchers' experiences of methodologically incongruent peer review feedback. Qual Psychol. (2025) 12:7–24. 10.1037/qup0000322

[129] World Health Organization. World health statistics 2024: monitoring health for the SDGs, Sustainable Development Goals (2024c). Geneva: World Health Organization. Available online at: https://www.who.int/news/item/24-05-2024-COVID-19-eliminated-a-decade-of-progress-in-global-level-of-life-expectancy (Accessed June 11, 2025).

[130] Venuleo C Marino C Ferrante L Rollo S Schimmenti A. (2022). Internet use during the COVID-19 outbreak: a resource for well-being or an amplifier of psychological distress? A study on an Italian sample. Mediterr J Clin Psychol. 10. 10.13129/2282-1619/mjcp-3463

[131] Fancourt D Steptoe A Bu F. Trajectories of anxiety and depressive symptoms during enforced isolation due to COVID-19 in England: A longitudinal observation study. Lancet Psychiat. (2021) 8:141–9. 10.1016/S2215-0366(20)30482-X 33308420 PMC7820109

